# Systematic review and meta-analysis on the global distribution, host range, and prevalence of *Trypanosoma evansi*

**DOI:** 10.1186/s13071-019-3311-4

**Published:** 2019-01-31

**Authors:** Weldegebrial G. Aregawi, Getahun E. Agga, Reta D. Abdi, Philippe Büscher

**Affiliations:** 1 Werer Agricultural Research Center, Ethiopian Institute of Agricultural Research, Werer, Afar Ethiopia; 2 Food Animal Environmental Systems Research Unit, U.S. Department of Agriculture, Agricultural Research Service, Bowling Green, KY USA; 3 Department of Veterinary Biomedical Sciences, Long Island University, Greenvale, NY USA; 4 Department of Biomedical Sciences, Institute of Tropical Medicine, Antwerp, Belgium

**Keywords:** Trypanosomosis, *Trypanosoma evansi*, Surra, Meta-analysis, Systematic review, Prevalence, Camel

## Abstract

**Background:**

Surra is an animal trypanosomosis, caused by infection with *Trypanosoma evansi* and leading to severe economic loss due to mortality and morbidity. Compared to tsetse-transmitted animal trypanosomoses, little attention is given to the epidemiology and control of surra. Understanding its epidemiology is a first step in local and global efforts to control the disease. We conducted a systematic review and meta-analysis of published studies on distribution, host ranges and prevalence of *T. evansi* infection.

**Methods:**

Four electronic databases were searched for publications on *T. evansi* that met our inclusion criteria for the systematic review. Subsets of publications were subjected to meta-analysis for the pooled prevalence of *T. evansi* in various hosts as determined by multiple detection methods.

**Results:**

A total of 272 references published between 1906–2017 were included. *Trypanosoma evansi* was reported from 48 countries; largely confined to Africa and Asia with publications on natural *T. evansi* infections from 77% (*n* = 48) of countries, contrasting with seven countries in South America, and four in Europe where *T. evansi* is not endemic but was imported with infected animals. Although surra is a notifiable disease, many countries do not report surra cases to OIE. *Trypanosoma evansi* was mainly reported from dromedary camels in Africa and the Middle East, water buffaloes, cattle, dogs and horses in East and Southeast Asia. In South America, the acute form of the disease was reported in horses and dogs. Surra was also reported in a wide range of wild animals. Some rare human cases occurred in India and Vietnam. Meta-analysis on a subset of 165 publications indicated pooled prevalence of *T. evansi* in domestic animals ranging from 14–31%, 6–28% and 2–9% using respectively antibody detection, molecular and parasitological tests, with camels as the most affected, followed by buffalo and cattle.

**Conclusions:**

This study illustrates that *T. evansi* affects a wide range of domestic and wild animals in Africa, Asia and South America with highest prevalence observed in dromedary camels. For successful control of *T. evansi*, both locally and globally, the role of wild animals in the epidemiology of surra needs further investigation.

**Electronic supplementary material:**

The online version of this article (10.1186/s13071-019-3311-4) contains supplementary material, which is available to authorized users.

## Background

Trypanosomes are unicellular flagellar protozoa belonging to the family of Trypanosomatidae and the genus *Trypanosoma* [1]. The genus *Trypanosoma* comprises many species causing diseases called trypanosomoses in domestic and wild animals, as well as in humans [2]. Livestock trypanosomoses, caused by *Trypanosoma brucei*, *T. equiperdum* and *T. evansi* that all belong to the subgenus *Trypanozoon*, have a significant socio-economic impact, and limit animal productivity throughout the world [3]. *Trypanosoma evansi* was the first pathogenic mammalian trypanosome to be described in 1880 by Griffith Evans in the blood of Indian equines and dromedaries [4]. The species evolved from *T. brucei* by adaptation to mechanical transmission, enabling it to spread beyond the tsetse belt in Africa, causing a wasting disease of livestock commonly named “surra” in Asia and Africa, and “mal de cadeiras” in Brazil [4]. Among the pathogenic trypanosome species, *T. evansi* is known to infect a large diversity of mammalian hosts, including endangered wild animals. Its main difference from the other trypanosomatids is the lack of maxicircle kinetoplast DNA (kDNA). *Trypanosoma evansi* does not develop in its vector [4, 5]. It is mechanically transmitted by hematophagous flies from the genera *Stomoxys* and *Tabanus*. Its mechanical transmission depends on the survival of the parasites in the oral cavity of the vector. Consequently, the smaller the interval of vector blood-sucking between an infected and an uninfected animal, the greater the success of parasite transmission [4]. In South America, transmission can occur by the common vampire bat *Desmodus rotundus* during its blood meal, acting as both vector and host [6]. Oral transmission to carnivores when feeding on fresh infected meat or carcasses has been described as well [7, 8].

Surra and its causative agent, *T. evansi* are widely distributed throughout tropical and subtropical regions of Northern Africa, Southeast Asia, as well as Central and South America [9]. In Europe, the importation of infected dromedary camels from the Canary Islands caused outbreaks in France [10] and Spain [11]. Surra kills thousands of animals every year [12]. The course of infection ranges from an acute disease with high mortality to a chronic infection characterized by subcutaneous edema, fever, lethargy, weight loss, abortion, nasal and ocular bleeding, and stiffness of the limbs. Surra can lead to neuropathy and immune suppression coupled with anemia eventually leading to death in both domestic and wild mammals [3, 13–15]. Clinical signs of neurological disorders are reported in horses, camels, buffaloes, cattle, deer and cats infected by *T. evansi* [3]. Surra has been associated with failure in vaccination against important transboundary animal diseases such as foot and mouth disease, hemorrhagic septicemia and classical swine fever [16], which pose significant impacts on global trade in live animals and animal products. Recently, there have been reports of the zoonotic potential of *T. evansi* from India and Vietnam [17–20]. In 2009, the World Organization for Animal Health (OIE) classified surra as a notifiable multispecies animal disease [21].

Despite its economic and animal health impacts, surra has been severely neglected in terms of awareness, control interventions and research into improved control tools [22]. Although *T. evansi* has been studied over the past 100 years, the epidemiology of the disease remains hardly understood in many countries and funding agencies are blatantly ignorant on the impact of this disease on populations that depend on their domestic animals. In recent years, however, a growing number of investigations have been conducted on the prevalence of *T. evansi* infection among domestic and wild animals. To raise awareness about surra, an exhaustive literature review on the distribution of *T. evansi* and the economic losses that it causes, is the first step to take. The objective of this systematic review and meta-analysis study was to provide a global overview of the epidemiology of surra by assessing the geographical distribution of *T. evansi*, identifying domestic and wild animals that are naturally susceptible to the disease, and estimating the pooled prevalence of *T. evansi* in various animal host species.

## Methods

The systematic review (SR) and meta-analysis (MA) were conducted according to the Preferred Reporting Items for Systematic Reviews and Meta-Analyses (PRISMA) guidelines (Additional file 1: Table S1) [23]. Inclusion and exclusion criteria were defined in terms of the relevance of the references to achieve the study objectives.

### Literature search

A systematic search was conducted to identify all publications reporting the detection of *T. evansi* infection in any host. Four electronic databases - CAB Abstracts, Library of Institute of Tropical Medicine (EDS-ITM), PubMed, and ScienceDirect were searched using the search terms “evansi OR surra” applied in the title, abstract and the keywords, where applicable. No restrictions were applied with regards to language, location and date of publication (last search was run on August 17, 2017). Additional hand search of authors’ collections of relevant peer reviewed publications were also included. All references located in the searches were entered to RefWorks, a web-based reference manager software (ProQuest, Ann Arbor, MI, USA). Duplicate references with the same information about study location, numerator, denominator, and study period were removed, and abstracts were obtained for the remaining references.

### Relevant screening, inclusion and exclusion criteria

Initially, references were screened based on their titles. Unrelated references that were retrieved due to similarity in species names, such as *Lutzomyia evansi*, *Tetranychus evansi* and *Dipetalonema evansi*, were removed. In addition, references containing the term “experimental” in their title and confirmed to be exclusively about laboratory based experimental studies were removed. However, references about field trials and all ambiguous references were retained in the database for the next screening phase. All references with a title in a language other than English were stored in a dedicated RefWorks folder “Foreign language” for further screening.

References retained after initial screening were further scanned by abstract. If the information of the abstract was not sufficient to assess whether to include or remove a reference, the full text file of the publication was screened. Full text portable document format (PDF) files that were not freely accessible online were obtained through the library of the Institute of Tropical Medicine (ITM). Publications in Dutch, French, Portuguese, Spanish and Thai languages were handled by one of the authors (PB) and another colleague. Full text publications were screened according to the following inclusion criteria: (i) if a publication contained data on any positive diagnostic test result for *T. evansi* in any naturally infected host; (ii) if a publication contained data on incidence, prevalence, host range and distribution of *T. evansi* in any naturally infected host. References were excluded by abstract if *T. evansi* was not detected in any natural host by any diagnostic test. Full text publications were excluded for one or more of the following reasons: (i) diagnostic test not specified; (ii) sample source not described; (iii) literature review; (iv) publication reporting data published elsewhere; (v) case report based on clinical signs only; (vi) outbreak report without laboratory-based confirmation; (vii) reporting a zero prevalence in any diagnostic test; (viii) detection in horses where results are indistinguishable from *T. equiperdum* infection (dourine); (ix) publication exclusively on experimental infection. References were screened by two independent reviewers (WA and PB) with all disagreements resolved by consensus.

For the quantitative meta-analysis to estimate pooled prevalence, publications that contained relevant epidemiological information such as host species, sample size, diagnostic method and prevalence were retained. A priori defined inclusion criteria were set to include publications that provided applicable quantitative information on the epidemiology of *T. evansi*. Publications with case reports, samples collected after an outbreak of surra or from clinically sick animals, insufficient or unrepresentative sample, unclear report of sample size and prevalence reported on multiple species without stratified report of prevalence by species, were excluded from the meta-analysis. Prevalence estimation was carried out after categorization of diagnostic tests and some host species. Accordingly, diagnostic tests were combined into five categories: (i) parasitological methods include wet blood smear, stained blood smear, microhematocrit concentration, and mouse inoculation; (ii) antibody-based tests include antibody-based enzyme-linked immunosorbent assay (Ab-ELISA), card agglutination test for trypanosomiasis (CATT/*T. evansi*), complement fixation test (CFT), dipstick immunoassay (DIA), indirect fluorescence antibody test (IFAT), indirect hemagglutination (IHA), immune trypanolysis (ITL), LATEX, and reverse dot blot; (iii) antigen-based tests include antigen-based enzyme-linked immunosorbent assay (Ag-ELISA), LATEX-monoclonal antibody (LATEX-MAB), and Suratex; (iv) molecular tests include both regular and real time polymerase chain reaction (PCR) using different primers; and (v) non-specific immunological tests include formol gel test, mercuric chloride test, Takata reaction, and thymol turbidity. Species-wise categorization merged sheep and goat into “small ruminants”, horse, donkey and mule into “equine” and all studied wild animals into “wild animals”. Since large datasets were obtained for buffalo, cattle, dromedary camel, and dog analysis was carried out without categorization for these animal species.

### Data extraction

Reference information regarding author’s name, title, and year of publication were recorded in the data extraction file. From the included publications, data were extracted on country and study area (districts, province, region), duration of sample collection, host species, number of samples analyzed, type of samples collected, diagnostic method used, number of positives and prevalence or percentage. For publications that reported only the total number of animals sampled and the prevalence, the number of positives were calculated. Similarly, prevalence values were calculated for publications that reported only the number of samples and the number of positives. Case reports with the above information except prevalence data were also included for qualitative analysis. Data were extracted from the included publications by WA and PB, and any disagreement was discussed, and resolved. All data were recorded in an Excel spreadsheet (Microsoft Corp., Redmond, Washington, USA).

### Statistical analysis

For meta-analysis, descriptive statistics were applied to determine the total number of host species included at each level of analysis and the ranges of prevalence estimates. Random-effects meta-analyses were carried out (using the total sample size and number of positives) to estimate the prevalence of *T. evansi* in different hosts. Between-study variations were assessed using the Higgins I^2^ statistic to estimate the percentage of total variation in prevalence estimates across the studies attributable to heterogeneity rather than chance; I^2^ > 50% may indicate substantial heterogeneity [24]. Separate meta-analyses (subgroup analysis) were conducted on data subsets to estimate the pooled prevalence of *T. evansi* with various detection methods in different hosts stratified by country. The point estimates (with 95% confidence intervals) from separate datasets were pooled using the DerSimonian-Laird random effects method [25], with the variances of the raw proportions stabilized using the Freeman-Tukey double arcsine transformation [26, 27]. All meta-analyses were carried out using the “metaprop_one” routine in STATA version 15 (StataCorp. LLC, College Station, TX, USA).

## Results and discussion

**Fig. 1:**
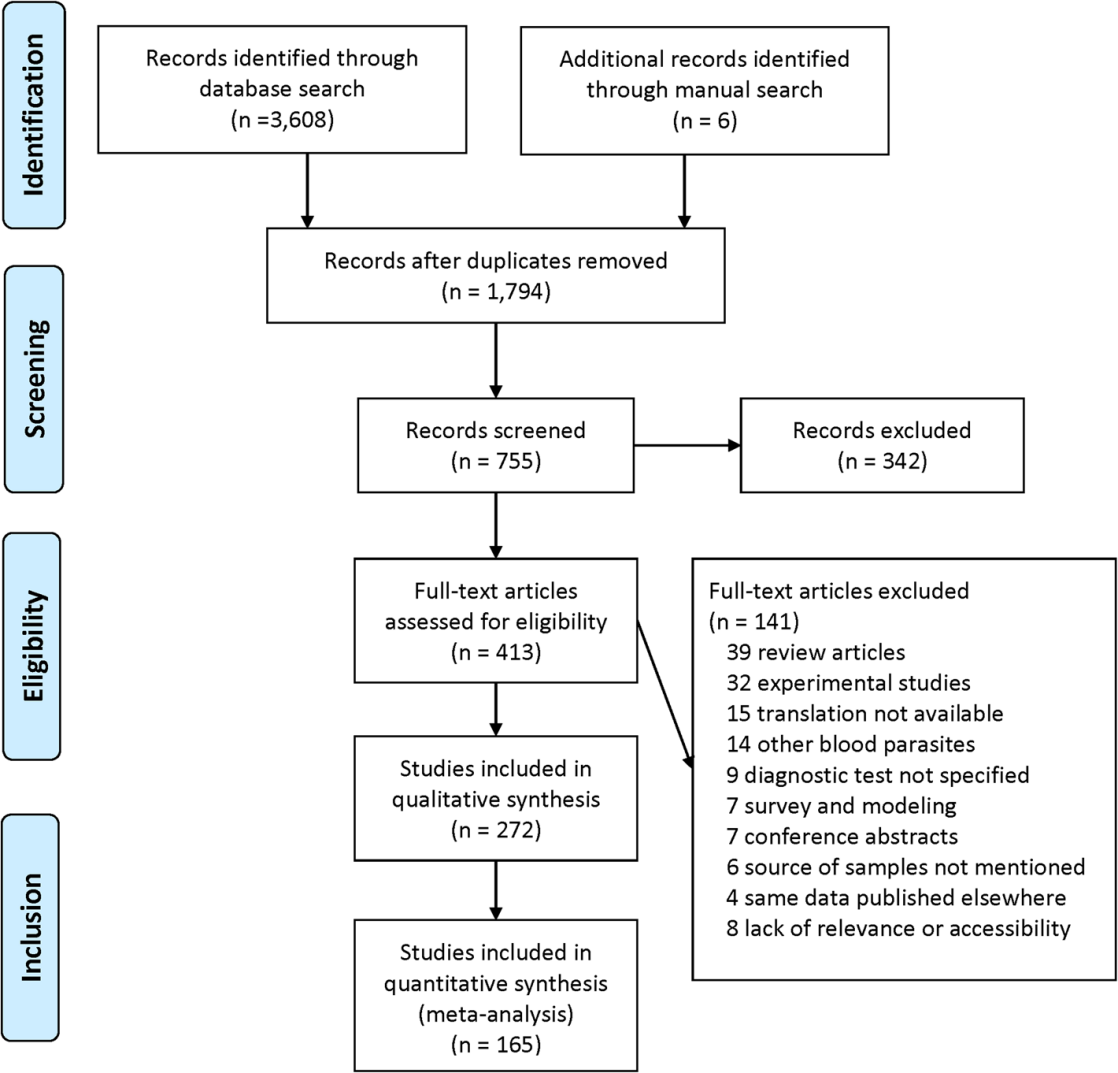
Flow chart representing the selection of studies for inclusion for the systematic review and meta-analysis of *Trypanosoma evansi* prevalence, geographical distribution and host range

From the initial searches based on reference titles, 3614 (3608 from databases, 6 from hand-search) potentially relevant publications were identified (Fig. 1). After primary screening of titles and abstracts, and duplicate removal, 413 references were selected for full text search. A total of 272 relevant publications that satisfied our inclusion criteria for SR were identified, of which two were in French, eight in Portuguese, two in Spanish, two in Thai, and the remaining 258 in English. Of the 272 publications selected for qualitative analysis, 165 (representing 399 datasets) were included in the MA for prevalence estimation.

**Fig. 2:**
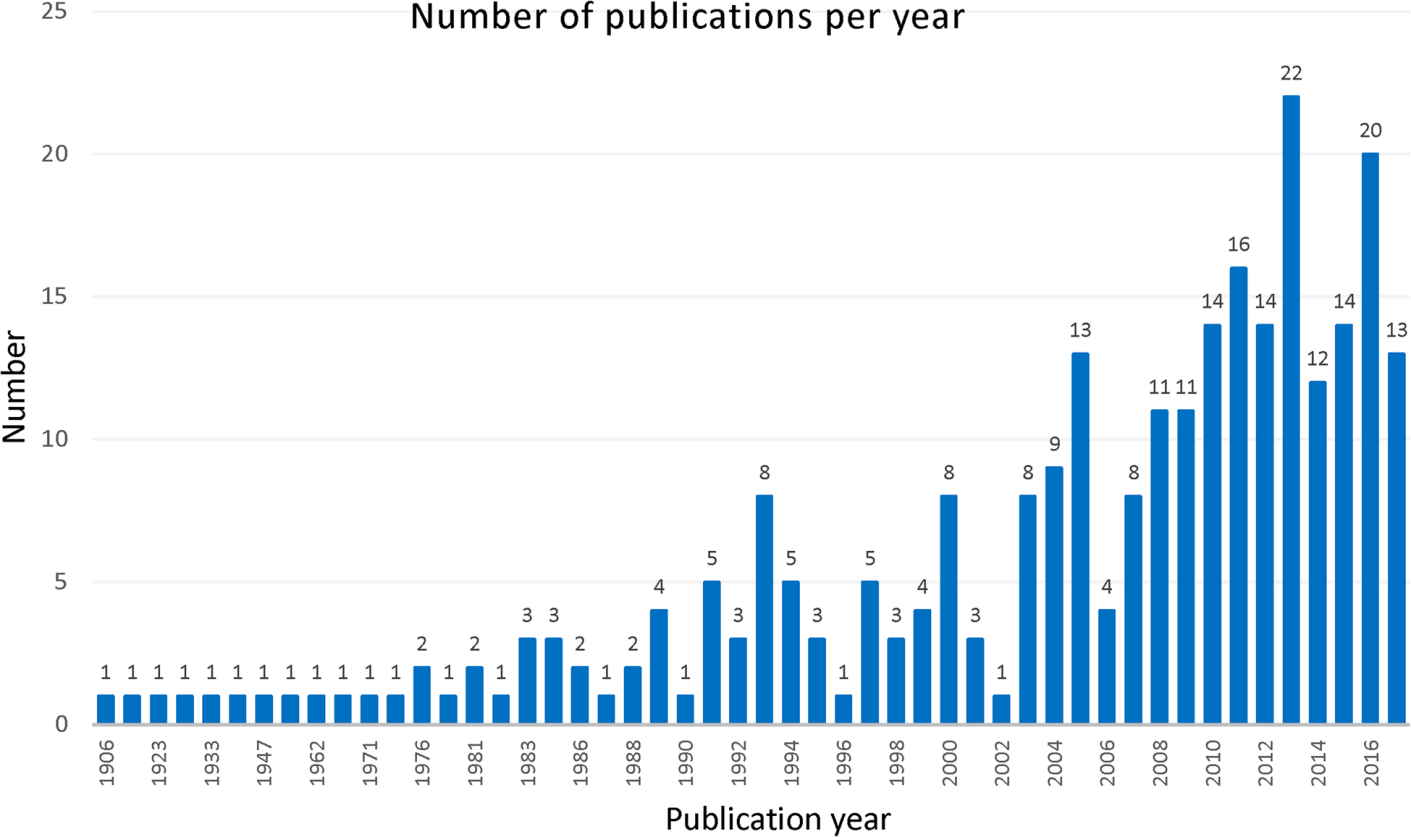
Number of publications included in the systematic review and meta-analysis of *Trypanosoma evansi* from 1906 to 2017

The distribution of included articles as a function of publication year is presented in Fig. 2. The oldest publication was dated from 1906. Only 29 publications were published between 1906 and 1988 with many years without any publication. Since 1990, the number of publications on surra increased slightly and a total of 42 references were included with an average of 3.8 per year between 1989 and 1999. Since 2000, the number of publications increased considerably and 201 references were included with an average of 11.2 per year during 2000–2017. In most publications after 2000, the use of multiple diagnostic tests on diverse host species was reported.

### Global distribution of surra

**Fig. 3:**
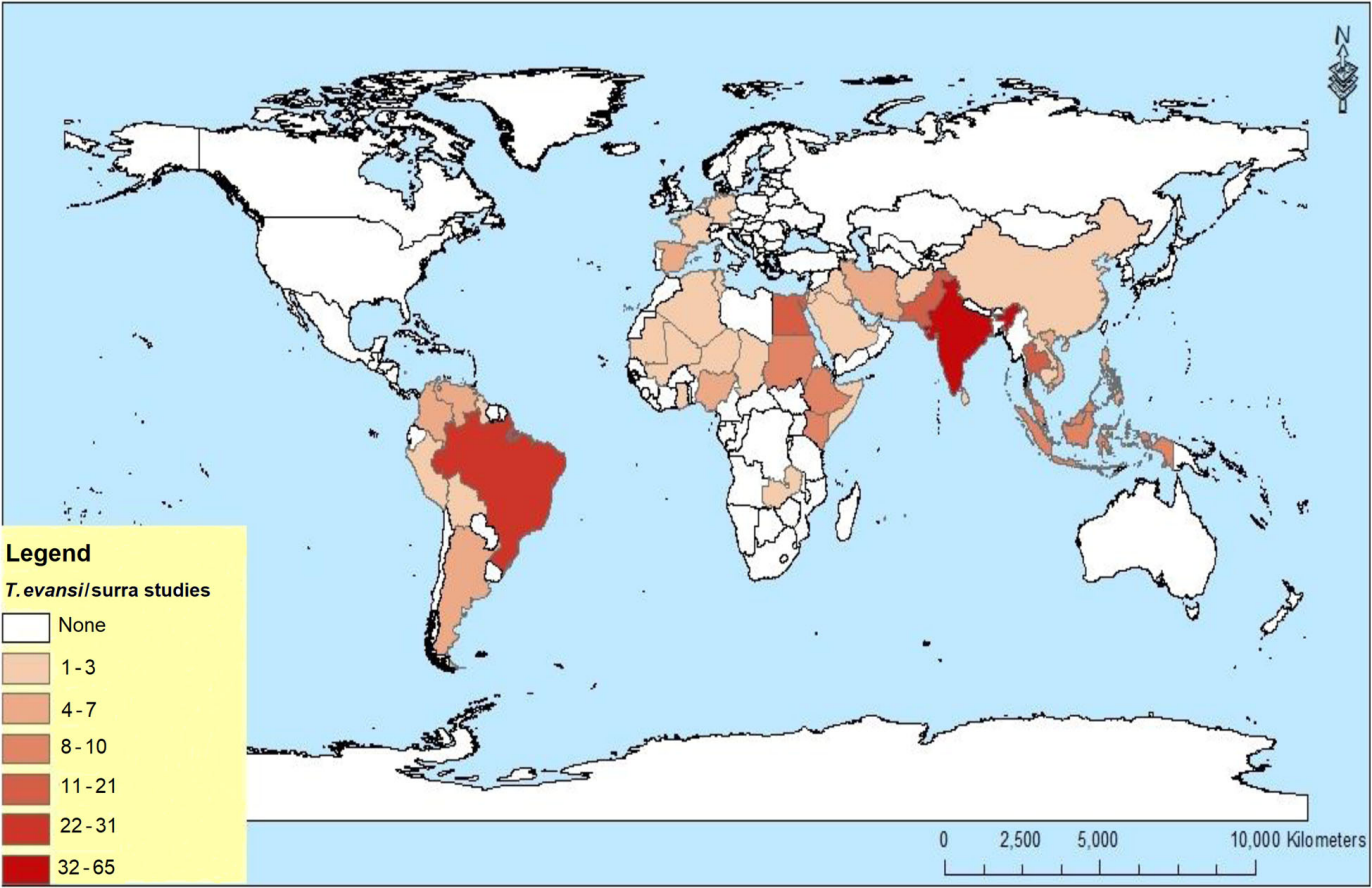
Global distribution of *Trypanosoma evansi* based on 272 studies published between 1906 and 2017 included in the systematic review

Natural infections with *T. evansi* were reported from 48 countries, including 20 in Asia, 17 in Africa, seven in South America, and four in Europe (Fig. 3, Table 1). No natural infections with *T. evansi* were reported in North America, Australia and Antarctica.

**Table 1:** Continents and countries with reported *T. evansi* infection in diverse host species and detected with diverse methods

Continent	Country	No. of studies	Host^c^	No. of studies per host	Detection methods	References
Africa	Algeria	2	Camel	1	Stained blood smear	[89]
			Horse	1	Unspecified microscopic method	[90]
	Chad	1	Camel	1	HCT and PCR	[91]
	Egypt	13	Buffalo	1	CATT	[82]
			Camel	10	Stained blood smear, mouse inoculation, CATT, ELISA, Suratex, Latex, thymol turbidity, PCR	[1, 77, 92–99]
			Cattle	2	ELISA, PCR	[100, 101]
			Goat	1	Stained blood smear, CATT, PCR	[96]
			Sheep	1	Stained blood smear, CATT, PCR	[96]
			Human	1	Stained blood smear, ELISA	[77]
	Ethiopia^a^	8	Camel	8	Stained blood smear, HCT, CATT, ITL, PCR	[83, 102–108]
			Cattle	1	HCT, CATT, PCR	[83]
			Donkey	1	CATT, ITL, PCR	[83]
			Goat	1	HCT, CATT, ITL, PCR	[83]
			Horse	1	PCR	[83]
			Mule	1	PCR	[83]
			Sheep	1	HCT, CATT, ITL, PCR	[83]
	Ghana^b^	1	Tsetse fly	1	PCR	[30]
	Kenya	9	Camel	9	HCT, mouse inoculation, CATT, ELISA, Suratex, Latex, IHA, PCR	[13, 109–116]
	Mali^a^	2	Camel	2	Unspecified microscopic method, ELISA	[48, 117]
	Mauritania^a^	2	Camel	2	Stained blood smear, CATT, IFAT	[118, 119]
	Mauritius	1	Deer	1	Stained blood smear	[120]
	Morocco	1	Camel	1	CATT, ELISA	[121]
	Niger^a^	1	Camel	1	HCT, CATT	[122]
	Nigeria	6	Camel	3	Stained blood smear, mouse inoculation	[123–125]
			Cattle	2	PCR	[126, 127]
			Horse	1	Stained blood smear	[128]
	Somalia^a^	3	Camel	3	Stained blood smear, HCT, ELISA	[129–131]
	Somaliland	1	Camel	1	CATT	[132]
	Sudan^a^	10	Camel	10	Stained blood smear, HCT, CATT, ELISA, IFAT, PCR	[88, 133–141]
			Goat	1	ELISA	[88]
			Sheep	1	ELISA	[88]
	Tunisia^a^	1	Dog	1	Stained blood smear, PCR	[142]
	Zambia^b^	1	Tabanids	1	PCR	[31]
Asia	Afghanistan	1	Dog	1	Stained blood smear, PCR	[143]
	Cambodia	1	Rodents	1	CATT, PCR	[63]
	China	1	Buffalo	1	Unspecified microscopic method	[144]
	India	65	Buffalo	16	Stained blood smear, mouse inoculation, ELISA, Latex, PCR	[86, 145–159]
			Camel	5	Stained blood smear, mouse inoculation, ELISA, Latex, PCR	[160–164]
			Cattle	17	Stained blood smear, mouse inoculation, ELISA, Latex, PCR	[86, 146, 149, 151, 153–156, 158, 165–172]
			Dog	13	HCT, stained blood smear, mouse inoculation, ELISA, Latex, PCR	[61, 173–184]
			Donkey	1	ELISA	[185]
			Elephant	1	Unspecified microscopic method	[186]
			Goat	1	Stained blood smear	[187]
			Horse	9	Stained blood smear, mouse inoculation, ELISA, CATT, PCR	[86, 185, 188–194]
			Human	5	Stained blood smear, mouse inoculation, CATT, PCR	[17–19, 195, 196]
			Jaguar	1	Stained blood smear	[7]
			Mule	1	ELISA	[185]
			Pig	1	Stained blood smear, mouse inoculation	[197]
			Pony	1	Stained blood smear	[198]
			Sheep	1	ELISA	[199]
			Tiger	2	Stained blood smear	[7, 200]
			Cattle and buffalo	2	Stained blood smear, ELISA	[201, 202]
			Cattle, buffalo and equine	1	Stained blood smear	[151]
			Horse and mule	1	Unspecified microscopic	[203]
			Equines	3	Stained blood smear, PCR	[151, 155, 201]
	Indonesia^a^	10	Buffalo	4	HCT, mouse inoculation, ELISA, CATT	[204–208]
			Cattle	8	HCT, mouse inoculation, ELISA, CATT, PCR	[204–206, 209–213]
			Horse	1	ELISA	[206]
	Iran^a^	6	Camel	6	Stained blood smear, PCR	[214–219]
	Iraq^a^	1	Cattle	1	Stained blood smear	[220]
	Israel^a^	1	Horse	1	CATT, reverse dot blot	[87]
	Jordan^a^	2	Camel	2	Stained blood smear, mouse inoculation	[85, 221]
			Horse	1	Unspecified microscopic method	[85]
	Kuwait	1	Camel	1	Stained blood smear	[222]
	Laos	2	Rodents	3	CATT, PCR	[63, 223]
	Malaysia	9	Cattle	2	HCT, stained blood smear CATT, mouse inoculation	[224, 225]
			Buffalo, cattle	1	CATT	[226]
			Deer	2	HCT, CATT	[227, 228]
			Dog	1	Stained blood smear	[229]
			Horse	3	HCT, stained blood smear, CATT, PCR	[224, 230, 231]
			Rhinoceros	1	Stained blood smear	[232]
	Pakistan^a^	14	Bear	2	Stained blood smear, PCR	[233, 234]
			Buffalo	2	Stained blood, PCR	[235, 236]
			Camel	5	Stained blood smear, CATT, ELISA, Suratex, PCR	[79, 237–240]
			Dog	1	Stained blood smear	[241]
			Horse	1	HCT, CATT, PCR	[84]
			Equines	3	Stained blood smear, unspecified microscopic method	[242–244]
	Palestine	1	Camel	1	Unspecified microscopic method	[245]
			Mule	1	Unspecified microscopic method	[245]
	Philippines^a^	6	Buffalo	4	Mouse inoculation, LAMP, PCR	[246–249]
			Cattle	2	PCR	[250, 251]
	Saudi Arabia	3	Camel	3	Stained blood smear, ELISA, IHA, PCR	[252–254]
	Sri Lanka	1	Dog	1	Mouse inoculation	[255]
	Thailand^a^	21	Buffalo	3	HCT, stained blood smear, mouse inoculation, ELISA and CFT	[51, 256, 257]
			Cattle	9	HCT, stained blood smear, mouse inoculation, ELISA, IFAT, Dipstick colloidal dye immunoassay (DIA) and PCR	[50, 52, 258–264]
			Deer (hog & rusa)	2	HCT, stained blood smear, mouse inoculation, ELISA	[265, 266]
			Dog	1	Unspecified microscopic method	[267]
			Elephant	1	PCR	[268]
			Pig	1	HCT, ITL	[269]
			Rodents	3	CATT, PCR	[63, 223, 270]
			Horse and mule	1	HCT, stained blood smear and mouse inoculation	[53]
	United Arab Emirates^a^	1	Camel	1	Stained blood smear, ELISA	[271]
	Vietnam	4	Buffalo	3	HCT, mouse inoculation, CATT, LATEX, ELISA, ITL, PCR	[54, 272, 273]
			Human	1	Stained blood smear, CATT, PCR	[20]
South America	Argentina	4	Capybara	2	Stained blood smear, PCR	[66, 274]
			Dog	1	Stained blood smear, PCR	[56]
			Horse	1	Unspecified microscopic method, ELISA	[275]
	Bolivia^a^	3	Cattle	3	CATT, PCR	[12, 276, 277]
	Brazil^a^	30	Armadillos	1	PCR	[71]
			Bats (nectar feeding & vampire)	2	PCR	[59, 71]
			Buffalo	2	PCR	[37, 71]
			Capybara	4	HCT, CATT, IFAT, ELISA, PCR	[58, 65, 71, 278]
			Cattle	3	HCT, ELISA, PCR	[37, 58, 71]
			Crab-eating fox	1	HCT, IFAT	[62]
			Coatis	5	HCT, IFAT, PCR	[71, 72, 279–281]
			Deer	2	PCR	[73, 282]
			Dog	12	HCT, stained blood smear, mouse inoculation CATT, IFAT, ELISA, PCR	[55, 58, 62, 71, 278, 279, 283–288]
			Gray brocket	1	PCR	[73]
			Horse	12	HCT, stained blood smear, mouse inoculation, CATT, IFAT, ELISA, PCR	[38, 55, 57–59, 71, 279, 289–293]
			Ocelot	1	HCT, IFAT	[62]
			Marsupials	3	HCT, IFAT, PCR	[59, 71, 294]
			Peccaries (white-lipped & collared)	3	IFAT, PCR	[59, 70]
			Pig (feral)	2	IFAT, PCR	[59, 70]
			Crab-eating raccoon	1	PCR	[72]
			Rodents	4	HCT, IFAT, PCR	[59, 71, 74, 294]
	Colombia	7	Bat (vampire)	2	Stained blood smear, PCR	[68, 295]
			Capybara	2	Stained blood smear	[64, 296]
			Cattle	1	PCR	[297]
			Dog	2	Stained blood smear, PCR	[36, 298]
	Guyana	1	Sheep	1	IFAT	[299]
	Peru	2	Capybara	1	Unspecified microscopic method	[300]
			Cattle	1	PCR	[12]
	Venezuela^a^	5	Bat (nectar-feeding)	1	PCR	[69]
			Capybara	3	HCT, IFAT, PCR	[301–303]
			Cattle	2	HCT, PCR	[304]
			Donkey	1	HCT	[302]
			Horse	1	HCT	[302]
Europe	France	1	Camel	1	Unspecified microscopic method, mouse inoculation, CATT, ELISA, PCR	[10]
	Germany	1	Dog	1	Stained blood smear, CATT, PCR	[41]
	The Netherlands	1	Dog	1	Unspecified microscopic method	[42]
	Spain	6	Camel	5	HCT, Stained blood smear, CATT, ELISA, PCR	[11, 305–308]
			Cattle	1	CATT	[39]
			Donkey	1	CATT, PCR	[11]
			Goat	1	CATT	[39]
			Horse	1	HCT, CATT, PCR	[11]
			Sheep	1	CATT	[39]
			Equines	1	Unspecified microscopic method, CATT	[308]

^a^Reports occurrence of the disease to OIE

^b^Doubtful evidence obtained by non-*T. evansi* specific PCR

^c^Scientific names of wild animals are provided in Table 2

**Fig. 4:**
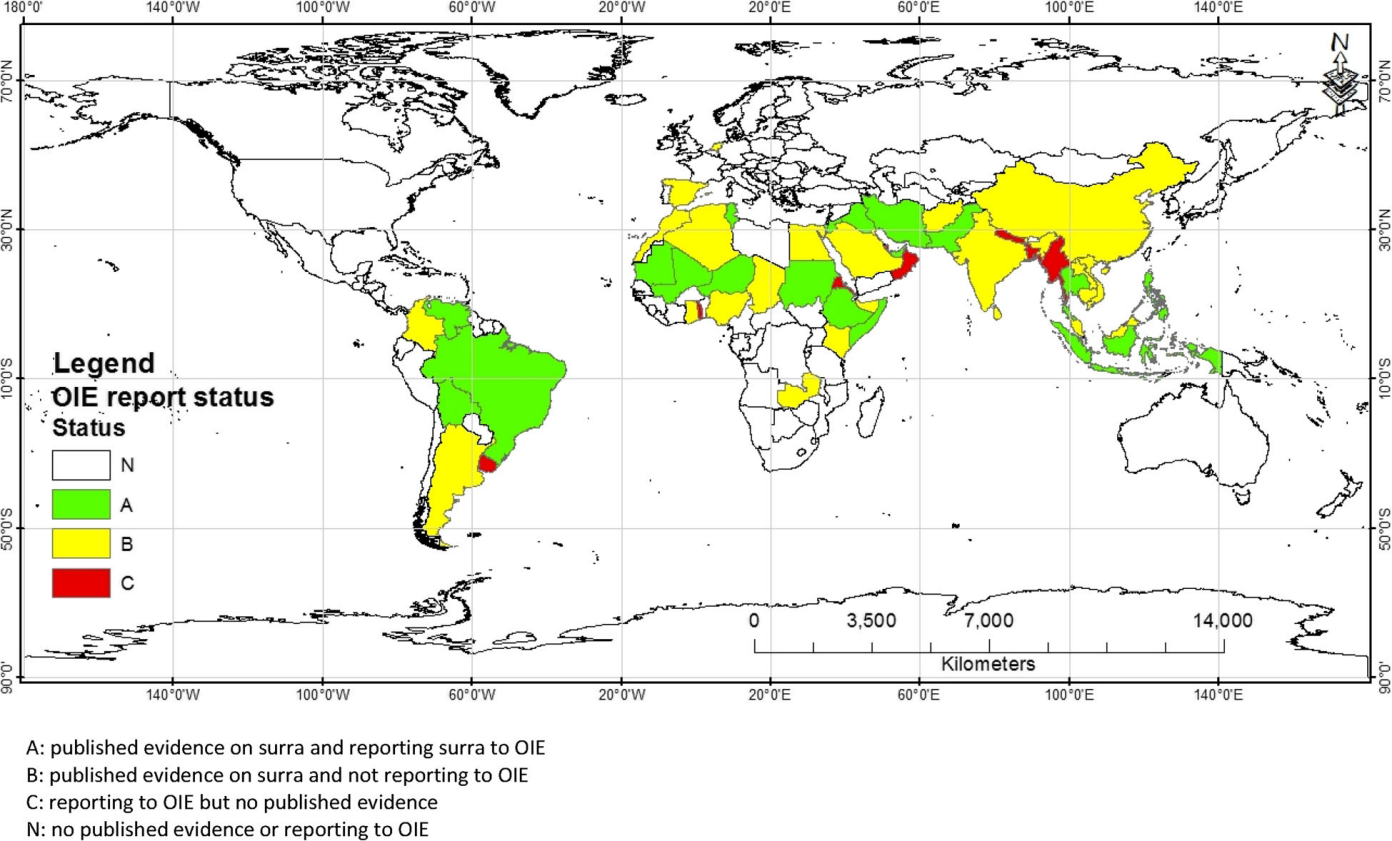
World map showing discordance between countries reporting surra to OIE and geographical distribution of surra based on published literature

Since surra became an OIE notifiable disease in 2009, 27 countries reported the presence of the disease at least once. Eight of these countries (Bangladesh, Eritrea, Myanmar, Nepal, Oman, Qatar, Togo, Uruguay) were not represented in the publications that we retrieved for this systematic review (Fig. 4). The geographical distribution of surra might not be limited to the present findings since our study included only publications with original data (excluding review articles). In addition, only natural infections of *T. evansi* with laboratory confirmations were considered. As with any systematic review, limitations associated with selection bias should considered in this study. For example, despite several studies indicating the widespread occurrence of surra in the southern part of China as reviewed by Lun et al. [28], only one publication was included in this review probably due to language and limitations in translation. Furthermore, we only searched four globally recognized databases probably missing publications which may have been equally relevant to this study.

Surra originated in Africa where *T. evansi* evolved from *T. brucei brucei* by partial (maxicircles) or complete (mini- and maxicircles) loss of kinetoplastic DNA [5, 29]. Sixty-three publications reported *T. evansi* infections in 17 African countries (Table 1). However, the presence of *T. evansi* in Ghana and Zambia is not fully supported by the data presented in the corresponding publications [30, 31]. In these two countries, *Trypanozoon* DNA was detected by PCR (ITS1) and sequencing of the amplicons were suggestive of *T. evansi* but ITS1 sequences contain polymorphisms that are shared among all *Trypanozoon* taxa. Isolation of *T. evansi* type B which typically lacks the *RoTat 1.2* gene parasite was reported from Kenya and Ethiopia [22, 32]. According to the narrative review of Desquesnes and co-workers [3], *T. evansi* was also reported from Libya and Burkina Faso. However, our literature search did not retrieve references for these countries.

It is generally accepted that *T. evansi* has spread from Africa into Asia through infected host species, particularly dromedary camels, horses and mules [4]. Analysis of historical data however suggests that surra was already present in India since time immemorial, at least VIII centuries B.C. [3]. The species *T. evansi* was first described as a parasite isolated from a horse in India [4, 33]. One hundred forty-eight publications described the occurrence of *T. evansi* in 20 Asian countries (Table 1). Although the majority of the retrieved publications on surra in Asia were from India, this and 10 other Asian countries did not report the disease to OIE since reporting started in 2009. Many possible explanations may exist for non-reporting of surra to the OIE, of which lack of awareness of its economic impact may be one. Another reason may be the usually chronic nature of the disease when it has become endemic. Also, we cannot exclude that countries refrain from reporting by fear of the consequences for trade of livestock and livestock products. The narrative review of Desquesnes et al. [3] mentions surra in Bhutan, Kazakhstan, Mongolia, Russia and Syria but our literature search did not retrieve any references on these countries that also did not report to OIE.

In South America, *T. evansi* was probably introduced during the 16th century with infected horses or mules of the Spanish conquistadores [34, 35]. With a total of 52 publications, the presence of *T. evansi* was reported in seven South American countries namely, Argentina, Bolivia, Brazil, Colombia, Guyana, Peru and Venezuela. Although our literature search did not retrieve any reference, *T. evansi* was reported to occur in Panama by Jaimes-Dueñez et al. [36]. More than half (30) of the publications were from Brazil, mainly from the Pantanal and Mato Grosso do Sul regions. In Pantanal, a vast flood plain in the center of South America, *T. evansi* is enzootic, infecting both domestic and wild animal species with different infective competencies [37, 38].

Nine publications reported *T. evansi* infections in Europe, all imported from non-European endemic countries. Six were about the Canary Islands that belong to Spain, where the disease became endemic after the import of dromedary camels in 1997 [39]. It is thought to have been imported there by illegal introduction of camels from Mauritania or Morocco [3]. Despite this published evidence, *T. evansi* has not been included in the animal health conditions for international trade within the European Union and other countries, resulting in two surra outbreaks originating from Gran Canaria. The first one occurred in metropolitan France (Aveyron) in 2006 in a camel farm, and the other occurred in metropolitan Spain (Alicante) in 2008 in a mixed camel and horse farm [10, 11]. Both outbreaks were controlled by containment and treatment of all suspected and confirmed cases and surveillance of animals that were in contact with the outbreak animals. Both outbreaks that had occurred before 2009 were reported to OIE. Recently, a new document on the assessment of *T. evansi* infection including surra was developed with the framework of European animal health regulation [40]. In Germany and the Netherlands, the disease was observed in two dogs with respectively a travel history to Brazil, Spain and Thailand, and to Nepal [41, 42]. *Trypanosoma evansi* was also suspected to occur in Turkey and in Bulgaria [3] although no references were retrieved for these countries.

In Oceania, CATT/*T. evansi* seropositive animals (cattle, pig and wallaby) were observed in the Irian Jaya, a border area of Papua New Guinea with Western Indonesia but were not confirmed by parasitological or molecular tests [43]. Nevertheless, the potential role of Timor rusa deer (*Rusa timorensis*) to spread *T. evansi* into Papua New Guinea must be considered [44]. Since European settlement, five exotic trypanosomes (*T. lewisi*, *T. melophagium*, *T. theileri*, *T. nabiasi* and *T. evansi*) have been identified in Australia from the various introduced mammals [45]. Fortunately, the surra-infected dromedary camels that were imported into Australia in 1907 were diagnosed quickly and *T. evansi* was eradicated from Australia before it spread [46]. Ever since, important efforts were made to prevent *T. evansi* from entering Australia and having a devastating effect on livestock and wild animals, including native marsupials that are highly susceptible to infection [43, 47].

### Host range of *T. evansi*

Our literature review confirms the very large host range of *T. evansi* that can naturally parasitise almost all domestic and many wild mammalian hosts (Table 2). *Trypanosoma evansi* was reported from dromedary camel (hereafter ‘camel’), equines, cattle, goat and sheep, water buffalo (hereafter ‘buffalo’), dog and pig. Apart from the host species identified in our review, *T. evansi* has also been reported to naturally infect domestic cat, bactrian camel and llama [40].

**Table 2:** Host range of *T. evansi* according to the 272 publications included in the systematic review

Hosts	No. of publications^a^	No. of countries	List of countries
Domestic animals			
Camel	83	23	Algeria, Chad, Egypt, Ethiopia, France, India, Iran, Jordan, Kenya, Kuwait, Mali, Mauritania, Morocco, Niger, Nigeria, Pakistan, Palestine, Saudi Arabia, Somalia, Somaliland, Spain, Sudan, United Arab Emirates
Cattle	57	16	Bolivia, Brazil, Colombia, Egypt, Ethiopia, India, Indonesia, Iraq, Malaysia, Papua New Guinea, Nigeria, Peru, Philippines, Spain, Thailand, Venezuela
Buffalo	37	10	Brazil, China, Egypt, India, Indonesia, Malaysia, Pakistan, Philippines, Thailand, Vietnam
Horse	37	14	Algeria, Argentina, Brazil, Ethiopia, India, Indonesia, Israel, Jordan, Malaysia, Nigeria, Pakistan, Spain, Thailand, Venezuela
Dog	34	12	Afghanistan, Argentina, Brazil, Colombia, Germany, India, Malaysia, Netherlands, Pakistan, Sri Lanka, Thailand, Tunisia
Equines	7	3	India, Pakistan, Spain
Sheep	6	6	Egypt, Ethiopia, Guyana, India, Spain, Sudan
Goat	5	5	Egypt, Ethiopia, India, Spain, Sudan
Donkey	4	4	Ethiopia, India, Spain, Venezuela
Pig	3	3	India, Thailand, Papua New Guinea
Mule	3	4	Ethiopia, India, Palestine, Thailand
Pony	1	1	India
Wild animals			
Capybara (*Hydrochoerus hydrochaeris*)	12	5	Argentina, Brazil, Colombia, Peru, Venezuela
Rodents (Rodentia)	10	4	Brazil, Cambodia, Laos, Thailand
Deer (Cervidae)	8	4	Brazil, Malaysia, Mauritius, Thailand
Bats (Chiroptera)	5	3	Brazil, Colombia, Venezuela
Coatis (*Nasua nasua*)	5	1	Brazil
Peccaries (Tayassuidae)	3	1	Brazil
Marsupials (Marsupialia)	3	1	Brazil
Bear (*Ursus thibetanus*)	2	1	Pakistan
Elephant (*Elephas maximus*)	2	2	India, Thailand
Feral pig (*Sus scrofa*)	2	1	Brazil
Tiger (*Panthera tigris*)	2	1	India
Armadillo (Dasypodidae)	1	1	Brazil
Crab-eating fox (*Cerdocyon thous*)	1	1	Brazil
Jaguar (*Panthera onca*)	1	1	India
Ocelot (*Leopardus pardalis*)	1	1	Brazil
Crab-eating raccoon (*Procyon cancrivorus*)	1	1	Brazil
Sumatran rhinoceros (*Dicerorhinus sumatrensis*)	1	1	Malaysia
Agile wallaby (*Macropus agilis*)	1	1	Papua New Guinea

^a^Multiple publications reported on multiple animal species

Of the domestic animals, camel is the most studied species (83 references) followed by cattle (57), horse (37), buffalo (37), and dog (34). Similarly, camel surra appears to have the widest geographical distribution being detected in 23 countries followed by cattle, horse and dog in 16, 13 and 12 countries, respectively. The principal host species of *T. evansi* varies among the different continents.

In Africa, major outbreaks of surra were reported in camel [48, 49] which corresponds with the fact that 13 of the 17 endemic African countries reported its occurrence in camels (Table 2). Our literature review shows that camel is the only host species reported with *T. evansi* infection in Chad, Kenya, Mali, Mauritania, Morocco, Niger, Somalia and Somaliland. It can therefore be concluded that among domestic animals in Africa, surra is mainly a disease of camels. In the camel, surra causes a great impairment of productivity and is considered the most economically important disease. It causes anorexia, weakness and emaciation that lead to low milk and meat yield, poor traction power, increased abortion and death [49]. Apart from camel, *T. evansi* in Africa was also reported from cattle, equine, small ruminants, dog and buffalo. Surra is generally considered a mild or negligible infection in cattle [50] although cattle, buffalo, pigs, goat and sheep that are infected with *T. evansi* suffer from immunosuppression, resulting in increased susceptibility to other diseases or in vaccination failure [16].

Also, in the Middle East camels appear to be the main affected domestic animal species. Outbreaks with clinical cases of camel trypanosomosis characterized by high mortality and abortion were reported from this region. Clinical cases, outbreaks and high prevalence of camel surra were also reported from India and Pakistan. In East and Southeast Asia, *T. evansi* mainly affects different breeds of buffaloes, cattle, dogs and horses. Apart from the high prevalence of the disease in these species, many outbreaks associated with abortion and still birth were reported [51–54]. Asia is the first region where cattle disease caused by *T. evansi* appears to be medically and economically important [50]. The pathogenicity of *T. evansi* seems to be diverse among the Southeast Asian countries, inducing fever, weight loss, nervous symptoms and abortion.

In South America, *T. evansi* was reported in horse, cattle, buffalo, dog and sheep. It is mainly characterized by acute, progressive and severe anemia in dogs and horses [36, 55–57]. The chronic form of the disease is characterized by intermittent fever, widespread subcutaneous edema, progressive anemia, blindness, lethargy, and hemostatic alterations. In the Brazilian Pantanal, one of the most important breeding cattle centers in the country, *T. evansi* is endemic and infects various domestic and wild animals. In this region, surra in horses is called “mal de cadeiras”’, characterized by anemia, immunosuppression, emaciation, severe neurological signs and death of non-treated animals. Consequently, severe economic losses occur given that horses are of pivotal importance in cattle ranching activities [57–59].

Our literature review reveals that *T. evansi* in wild animals is almost exclusively studied in Asia and South America. In Asia, many outbreaks associated with high morbidity and mortality were reported in Timor rusa deer (*Rusa timorensis*) and hog deer (*Axis porcinus*). In addition, clinical cases and mortality due to surra were reported in Asian or Himalayan black bear (*Ursus thibetanus*), Asian elephant (*Elephas maximus*), leopard (*Panthera pardus*), tigers (*Panthera tigris*), jaguar (*Panthera onca*) and Sumatran rhinoceros (*Dicerorhinus sumatrensis*) all considered as endangered wild species in Asia [40]. Bhaskararao et al. [60] reported an outbreak of *T. evansi* in circus tigers after feeding with infected meat. The prevalence of *T. evansi* in wild ruminants and the possibility of oral transmission must be regarded as a potential threat to wild carnivores, including endangered species [61, 62]. Various species of wild rodents in which the parasite was detected in Laos, Cambodia and Thailand, may play a reservoir role in the region [63]. Taken together, published evidence exists that *T. evansi* is a potential threat to wildlife in Asia. In South America, *T. evansi* was found in a variety of wild mammals with high prevalence values in the South American ring-tailed coatis (*Nasua nasua*) and especially in the capybara (*Hydrochoerus hydrochaeris*). *Nasua nasua* and *H. hydrochaeris* are considered as reservoirs of *T. evansi* and are regarded as sources of infection for domestic animals [64, 65]. The capybara is a large rodent found in tropical to temperate freshwater wetlands of South America [66]. This rodent species is reportedly infected with *T. evansi* in Argentina, Brazil, Colombia, Peru and Venezuela, while detection of the parasite in coatis was reported only from Brazil. Both capybara and coatis can develop similar clinical signs as seen in domestic animals; however, infected capybaras are usually asymptomatic while in coatis, clinical disease with symptoms including depression, weakness, lethargy, and some degree of anemia have been described [15, 65, 67]. Also, in South America the common vampire bat (*Desmodus rotundus*) is known to transmit *T. evansi* to other animals when taking their blood meal from them. Apart from being a vector, vampire bats can succumb to the infection and can transmit the infection among themselves, thus functioning as a reservoir [6, 68]. One publication reported on the indirect evidence of *T. evansi* in a nectar feeding bat (*Leptonycteris curasoae*), a feeding habit that precludes direct transmission of the parasite to other animals [69]. Collared peccary (*Tayassu tajacu*), white-lipped peccary (*Tayassu pecari*) and feral pigs (*Sus scrofa*) in the Pantanal region may also play a role as maintenance host for *T. evansi* due to their cryptic infections (only detectable by PCR) associated with high seroprevalence values [70]. Similarly, *T. evansi* was detected only by PCR in blood samples of armadillos (*Euphractus* spp.), gray brocket (*Mazama gouazoubira*), crab-eating raccoon (*Procyon cancrivorus*) in Brazil [71–73]. Other references report *T. evansi* detection in pampas deer (*Ozotocerus bezoarcticus*), marsh deer (*Blastocerus dichotomus*), ocelot (*Leopardus pardalis*), marsupials and rodents, the latter in the Brazilian Pantanal. Except for capybaras and vampire bats, the role of the diverse wild animal species in the epidemiology of *T. evansi* is unknown [6, 64, 66, 68, 74].

Despite the large number of publications on this topic, *T. evansi* occurring in wild animal species in Asia and South America is seldom reported to OIE. For six countries reporting to OIE the presence of *T. evansi* in wild animals (Bangladesh, Bolivia, Myanmar, Mali, Nepal, Togo) we did not retrieve any publication in our literature search. Regarding Africa, we retrieved only one early publication on *T. evansi* in a deer in Mauritius. This reflects the poor attention that is paid to the potential role of wild animals as reservoirs of *T. evansi* in Africa, in contrast to the many studies on tsetse transmitted trypanosomes in wild animals in the continent [75, 76]. Nevertheless, wild animals may play a role on the epidemiology of *T. evansi* in Africa and in the rest of the world since under experimental conditions many wild host species are fully receptive and susceptible to *T. evansi* infection [3, 40].

Recently, three human cases with confirmed *T. evansi* infection were reported raising concerns about its zoonotic potential in endemic regions. Three publications describe infection of *T. evansi* in human patients, two from India [17, 18] and one from Vietnam [20] where diagnosis was confirmed by parasitological, molecular, and serological identification of the parasites. Also, all three cases were reported to World Health Organization (WHO) from which the drugs for treatment were obtained. The publication on a human case in Egypt [77] does not provide sufficient evidence that the patient was actually infected with *T. evansi*. Despite the wide host range of *T. evansi* in South America, human cases have not yet been reported in that continent. Compared to the closely related parasites of humans *T. brucei* (sleeping sickness) and *T. cruzi* (Chagas disease), less attention was given to possible *T. evansi* infections in humans. A recent report explains how *T. evansi* can infect humans that have a genetic or metabolic deficiency in the production of human trypanocide apolipoprotein L1 (APOL1) that is a trypanocidal component of normal human serum [17]. The patient from Vietnam did not have APOL1 deficiency when serum was tested after treatment. A transient insufficiency in APOL1 can however not be excluded [20].

### Prevalence of *T. evansi* in host animals and countries

A total of 165 publications (representing 399 datasets or studies) were included in the meta-analysis to estimate the prevalence of *T. evansi*. The datasets represented 152 parasitological, 114 antibody-based detection, 96 molecular, 27 antigen-based detection, and 10 non-specific immunological tests. Over one-third of the publications (143) were on camels followed by cattle (64). The characteristics of the included datasets, together with the pooled prevalence of *T. evansi* in various animal host species across all countries, stratified by detection method are presented in Table 3. Moreover, the pooled prevalence of *T. evansi* for all animal species, stratified by detection method and country are represented in Table 4.

**Table 3:** Pooled prevalence of *T. evansi* in various animal host species stratified by detection method

Host species	Detection method	No. of publications	No. of datasets	No. of animals tested	No. of animals positive	Pooled prevalence (95% CI)	Heterogeneity	
							I^2^ (%)	*P*-value
Buffalo	Parasitological	14	16	10,325	474	4 (7–11)	97.8	<0.01
	Ab-based	8	10	4425	1001	28 (19–37)	97.5	<0.01
	Ag-based	3	4	3003	1612	50 (33–67)	97.6	<0.01
	Molecular	13	13	2917	379	28 (17–41)	97.7	<0.01
Camel	Parasitological	52	73	37,565	2704	9 (7–11)	97.2	<0.01
	Ab-based	25	32	24,930	7069	31 (25–37)	99.0	<0.01
	Ag-based	10	13	5102	1546	26 (19–33)	96.9	<0.01
	Molecular	10	15	6093	1582	23 (15–32)	98.2	<0.01
	NS immunoglobulin	6	10	3683	1024	35 (23–48)	98.2	<0.01
Cattle	Parasitological	18	19	13,078	253	4 (2–6)	95.6	<0.01
	Ab-based	14	15	7981	1693	19 (10–29)	99.1	<0.01
	Ag-based	6	7	1229	461	40 (20–63)	98.4	<0.01
	Molecular	22	23	6860	851	16 (10–23)	98.2	<0.01
Dog	Parasitological	8	12	8118	89	2 (1–4)	91.7	<0.01
	Ab-based^a^	4	5	376	78	21 (12–33)	83.3	<0.01
	Ag-based^a^	1	1	70	5	–	–	–
	Molecular	2	2	181	37	–	–	–
Equine	Parasitological	9	10	2772	67	2 (1–5)	91.3	<0.01
	Ab-based	11	17	7397	1123	19 (11–27)	98.5	<0.01
	Ag-based^a^	1	1	364	15	–	–	–
	Molecular	7	10	2190	165	6 (1–14)	97.0	<0.01
Small ruminants	Parasitological^a^	1	2	445	2	–	–	–
	Ab-based	5	10	3059	458	14 (5–27)	98.7	<0.01
	Molecular^a^	1	2	445	14	–	–	–
Wild animals	Parasitological	15	18	1426	201	15 (10–21)	88.1	<0.01
	Ab-based	10	20	2265	588	22 (13–32)	96.7	<0.01
	Molecular	15	29	2102	205	13 (8–18)	88.0	<0.01
	Ag-based^a^	1	1	50	11	–	–	–

*Abbreviations*: NS immunoglobulin, methods that target nonspecific immunoglobulins; CI, confidence interval; I^2^, between-study heterogeneity; *P*-value, Cochran’s Q (chi-square) test of between study heterogeneity

^a^Estimates for subgroup categories with fewer than four datasets were omitted

**Table 4:** Estimated pooled prevalence values of *T. evansi* in different countries stratified by detection method and host species

Host species	Country	Diagnostic method					
		Parasitological		Ab-based		Molecular	
		No. of datasets	Prevalence (95% CI)	No. of datasets	Prevalence (95% CI)	No. of datasets	Prevalence (95% CI)
Camel	Algeria	1	14 (9–22)	–	–	–	–
	Chad	1	5 (4–6)	1	31 (29–32)	–	–
	Egypt	7	12 (5–22)	2	28 (24–32)	4	32 (8–63)
	Ethiopia	11	8 (5–13)	5	17 (8–28)	4	12 (8–18)
	India	3	8 (7–10)	–	–	–	–
	Iran	4	4 (0–11)	–	–	1	0 (0–3)^a^
	Jordan	3	29 (7–59)	–	–	–	–
	Kenya	10	11 (5–18)	4	51 (30–72)	1	26 (23–30)
	Kuwait	2	2 (0–4)	–	–	–	–
	Mali	2	6 (5–8)	–	–	–	–
	Mauritania	2	1 (1–2)	3	22 (15–29)	–	–
	Morocco	–	–	2	16 (15–17)	–	–
	Niger	1	12 (10–14)	1	44 (41–48)	–	–
	Nigeria	3	12 (2–29)	–	–	–	–
	Pakistan	3	4 (0–13)	3	44 (40–48)	2	31 (29–33)
	Saudi Arabia	2	4 (2–6)	2	13 (9–17)	1	25 (20–32)
	Somalia	4	4 (2–6)	1	56 (50–63)	–	–
	Somaliland	–	–	1	26 (25–28)	–	–
	Spain	2	1 (1–2)	2	6 (5–8)	–	–
	Sudan	11	9 (5–15)	4	51 (41–62)	2	40 (36–44)
	United Arab Emirates	1	50 (40–60)	1	50 (40–60)	–	–
	Overall	73	9 (7–11)	32	31 (25–37)	15	23 (15–32)
Buffalo	Brazil	–			–	2	42 (32–53)
	Egypt	–	–	1	24 (19–30)	–	–
	India	7	5 (0–12)			6	51 (27–75)
	Indonesia	4	6 (3–9)	3	54 (42–66)	–	–
	Pakistan	2	4 (3–5)	–	–	2	8 (7–9)
	Philippines	1	68 (57–78)	–	–	2	3 (1–4)]
	Thailand	1	10 (9–12)	2	17 (15–18)	–	–
	Vietnam	1	2 (1–4)	4	17 (12–24)	1	6 (3–9)
	Overall	16	7 (4–11)	10	28 (19–37)	13	28 (17–41)
Cattle	Bolivia	–	–	1	40 (36–44)	3	5 (1–12)
	Brazil	–	–	2	3 (1–4)	2	8 (6–11)
	Colombia	–	–	–	–	1	9 (7–12)
	Egypt	–	–	1	42 (37–48)	1	30 (27–35)
	Ethiopia	1	7 (5–10)	1	37 (33–42)	1	6 (4–9)
	India	9	2 (1–5)	1	2 (1–3)	7	37 (17–59)
	Indonesia	2	2 (1–2)	3	40 (31–49)	1	61 (47–74)
	Iraq	1	1 (0–4)	–	–	–	–
	Nigeria	–	–	–	–	2	0 (0–1)^a^
	Peru	–	–	–	–	1	3 (2–6)
	Philippines	–	–	–	–	2	1 (0–2
	PNG	–	–	1	13 (10–18)	–	–
	Spain	–	–	1	5 (2–9)		
	Thailand	5	6 (2–13)	4	14 (8–23)	1	30 (18–45)
	Venezuela	1	18 (9–33)	–	–	1	26 (18–36)
	Overall	19	4 (2–6)	15	19 (10–29)	23	16 (10–23)
Equine	Brazil	3	3 (0–12)	6	41 (16–69)	3	12 (0–36)
	Ethiopia	–	–	2	5 (2–9)	3	13 (1–31)
	India	2	3 (2–6)	4	12 (9–16)	1	2 (1–5)
	Indonesia	–	–	1	2 (1–4)	–	–
	Israel	–	–	2	6 (5–8)	–	–
	Jordan	1	10 (5–18)	–	–	–	–
	Malaysia	1	1 (0–1)	1	14 (11–17)	1	1 (1–2)
	Nigeria	2	2 (1–4)	–	–	–	–
	Pakistan	1	1 (0–2)	1	14 (11–18)	2	1 (1–2)
	Overall	10	2 (1–5)	17	19 (11–27)	10	6 (1–14)
Small ruminants	Ethiopia	–	–	4	5 (0–14)	–	–
	Guyana	–	–	1	23 (17–30)	–	–
	India	–	–	1	10 (8–13)	–	–
	Spain	–	–	2	5 (4–6)	–	–
	Sudan	–	–	2	57 (52–61)	–	–
	Overall	2		10	14 (5–27)	2	3 (2–5)
Dog	Brazil	4	4 (1–7)	5	21 (12–33)	–	–
	India	7	2 (1–3)	–	–	–	–
	Malaysia	1	0 (0–0)	–	–	–	–
	Overall	12	2 (1–4)	5	21 (12–33)	–	–
Wildlife	Argentina	2	11 (3–20)	–	–	1	10 (5–20)
	Brazil	12	14 (8–22)	15	27 (19–35)	19	17 (13–20)
	Cambodia	–	–	1	10 (7–15)	1	3 (1–8)
	Colombia	1	24 (13–41)	–	–	1	2 (1–6)
	Laos	–	–	1	4 (2–9)	1	3 (1–8)
	Pakistan	1	10 (3–30)	–	–	1	25 (11–47)
	Peru	1	28 (17–42)	–	–	–	–
	Papua New Guinea	–	–	1	4 (1–10)		
	Thailand	–	–	1	1 (0–3)	4	1 (0–4)
	Venezuela	1	9 (7–11)	1	50 (46–54)	1	31 (23–41)
	Overall	18	15 (10–21)	20	22 (13–32)	29	13 (8–18)

^a^Zero pooled prevalence was estimated from studies with very low prevalence reports

As expected, the diagnostic method used has a major impact on reported prevalence with studies using parasitological methods reporting a very low prevalence in all the species compared to the other detection methods. This is due to the fact that a large proportions of infections (50–80%) in the field are chronic, and do not develop detectable levels of parasitemia [78]. Although parasitological tests are relatively cheap and fast, and are highly specific, their analytical sensitivity is rather low (parasitemia > 10^2^ parasites/ml) except for mouse inoculation which can become positive when parasitemia is < 10 parasites/ml. However, mouse inoculation is time consuming and presents ethical concerns by the use of live animals [40]. As surrogate of parasite detection, antigen detection tests are expected to be poorly sensitive for the same reasons as parasitological tests but also due to the presence of antigen-antibody complexes [79]. Yet, in buffalo, cattle and camel, prevalence values observed by antigen detection were higher than, or almost as high as prevalence values observed with the other tests particularly with antibody detection. A possible explanation is that the antigen detection tests are prone to non-specific reactions causing false positive results as is the case with non-specific immunoglobulin detection tests that are still routinely used for screening of surra in low-resource laboratories [79]. Non-specific immunoglobulin detection was only applied on dromedary and yielded the highest pooled prevalence (35%) (Table 3). Importantly, both the antigen detection and non-specific immunoglobulin tests are not recommended by OIE for diagnosis of surra, in contrast to parasitological, serological and molecular diagnostic methods (Chapter 2.1.17 of the OIE terrestrial manual) [80]. Pooled prevalence values observed with antibody detection tests tend to be higher than with molecular tests, probably due to the fact that detectable levels of antibodies may persist for 2 to 22 months after successful trypanocidal treatment [81, 82]. On the other hand, antibody detection tests might be negative in animals that are still in the incubation period [10]. Molecular tests are considered superior to parasite and antigen detections due to their ability in detecting pre-patent and chronic infections [79]. However, sensitivity and specificity of molecular tests vary as a function of the target sequence, primers and probes. Comparative evaluations of the various diagnostic tests for the detection of *T. evansi* are available elsewhere in the literature [40, 49, 71, 79, 80, 82–84].

Species-wise, higher estimated prevalence values were observed in camel followed by buffalo and cattle. However, the prevalence values within each species depend on the diagnostic method used and the geographical region covered by the reports, with a high heterogeneity observed among countries as a result (Tables 3 and 4). For example in camel, parasitological prevalence ranged from 1% in Spain and Mauretania to 50% in United Arab Emirates while the molecular prevalence ranged from 0% in Iran to 40% in Sudan. Similarly, parasitological prevalence in buffalo varied between 2% in Vietnam and 68% in The Philippines although these data were collected in only one publication in both countries. The overall molecular prevalence in buffalo was 28% with only 3% in The Philippines but 51% in India. In cattle the prevalence of surra was mainly studied in India and Thailand with a 2% and 6% pooled parasitological prevalence, respectively. One study in Venezuela reported an exceptionally high parasitological prevalence of 18% characteristic for an outbreak situation. Overall pooled molecular prevalence in cattle was 16% with two publications about Nigeria where the pooled prevalence was 0% to the highest pooled prevalence observed in India (37%). Horses are considered very susceptible to surra, associated with acute disease and high mortality [55, 57], while donkeys and mules are less susceptible to develop the disease [3, 53, 85]. In this study, prevalence values for equine are estimated from studies in horse, donkey and mule. Even though most of the studies were carried out in horses, the combined effect of donkey and mule seems to underestimate the prevalence of surra in horse under the equine category. Also important to note is that in some countries surra prevalence in horses, at population level is generally low, but at the farm level it can be very high within a short period of time when biting flies are abundant [86, 87]. Small ruminants might play a role as reservoir of *T. evansi*, e.g. in camel rearing areas of eastern Africa where small ruminants and camel are herded together. Yet, these animals are seldom considered which is obvious from the single publication reporting on the parasitological and molecular prevalence of surra in Ethiopian goats and sheep [83]. Reported seroprevalence values are generally low (up to 10%) except for an early study carried out in Sudan (57%). However, cross-reactions with other possibly non-pathogenic trypanosomes might have led to this higher prevalence since parasitological test revealed zero prevalence [88]. Cross-reactions or false positivity may also account for the rather high pooled seroprevalence (21%) recorded in dogs in Brazil. Dogs might be carriers of *T. evansi* for a short period before they succumb to the infection, however they are not considered as important reservoirs but rather as epidemiological dead-end hosts that can function as sentinel hosts in a given study area [3, 40]. Investigations of *T. evansi* in wildlife were mainly carried out in South America (Brazil). Meta-analysis showed overall 15% parasitological prevalence, 22% seroprevalence and 13% molecular prevalence estimates in wildlife.

In general, this review indicated that surra is endemic in Africa, Asia and South America. In Africa, the presence of other tsetse-transmitted trypanosomes seems to overshadow surra, thus scarce information is available on surra from wildlife and humans. *Trypanosoma evansi* infects multiple mammalian species through the bite of flies, bats and carnivores, exhibiting a wide spectrum of virulence levels in different host species with multiple clinical symptoms, indicating the presence of diverse reservoirs, complex epidemiology and economic impacts.

As with any systematic review, limitations associated with potential publication bias should be considered in this meta-analysis. Statistical evaluation of publication bias was not undertaken for various reasons where variability was obviously expected within and among diagnostic test categories, geography, breed of animals sampled, period of study etc. The summary estimates derived from the meta-analyses reflect a weighted average of the records and should not be interpreted as estimates of the national prevalence of the disease.

## Conclusions

This systematic review and meta-analysis study provides comprehensive information on the geographical distribution, host range and prevalence of surra worldwide. The results confirm the wide geographical distribution and a very large host range of *T. evansi* where it can naturally parasitize almost all domestic mammals and many wild animals, and even humans. The meta-analysis showed considerable variation in estimated prevalence values as a function of diagnostic tests, host species and geography. Surra was reported from Africa, South America, Asia and Europe and not from Oceania, and North and Central America. However, many endemic countries, based on published evidence, did not report the disease to the OIE, and vice versa. In addition to the economic importance of the disease in livestock production, its detection from many endangered wild animals is an alarming situation.

## Additional file

Additional file 1: **Table S1.** PRISMA 2009 checklist. (DOCX 26 kb)

## Abbreviations

Ab: Antibody
Ag: Antigen
CATT: Card Agglutination Test for Trypanosomiasis
CFT: Complement Fixation Test
DIA: Dipstick Immunoassay
ELISA: Enzyme Linked Immunosorbent Assay
HCT: Hematocrit Centrifugation Technique
IFAT: Indirect Fluorescence Antibody Test
IHA: Indirect Hemagglutination
ITL: Immune Trypanolysis
MA: Meta-Analysis
MAB: Monoclonal Antibody
OIE: Office International des Epizooties
PCR: Polymerase Chain Reaction
SR: Systematic Review
